# Present and Future of Mosquito-Borne Disease Control in Europe with a Specific Focus on the Mediterranean

**DOI:** 10.3390/insects17030254

**Published:** 2026-02-27

**Authors:** Maria Cholvi, Riccardo Moretti, Hugo Costa Osório, Gregory L’Ambert, Gonçalo Seixas, Mihaela Kavran, Antonios Michaelakis, Avgoustinos S. Stephanou, Christiana P. Antoniou, Angeliki F. Martinou, David Roiz, Maurizio Calvitti, Rubén Bueno-Marí

**Affiliations:** 1 Area of Parasitology, Department of Pharmacy and Pharmaceutical Technology and Parasitology, Faculty of Pharmacy, Universitat de València, 46100 Valencia, Spain; maria.cholvi@uv.es; 2 Department for Sustainability, ENEA (Italian National Agency for New Technologies, Energy and Sustainable Economic Development), 00123 Rome, Italy; maurizio.calvitti@enea.it; 3 Centre for Vectors and Infectious Diseases Research Doutor Francisco Cambournac (CEVDI), National Institute of Health Doutor Ricardo Jorge (INSA), 2965-575 Palmela, Portugal; hugo.osorio@insa.min-saude.pt; 4 Faculty of Medicine, Environmental Health Institute (ISAMB), University of Lisbon, Av. Prof. Egas Moniz, Ed. Egas Moniz, Piso 0, Ala C, 1649-028 Lisbon, Portugal; 5 Entente Interdépartementale pour la Démoustication Méditerranée, 34184 Montpellier, France; glambert@eid-med.org; 6 GIMM—Gulbenkian Institute for Molecular Medicine, Av. Prof. Egas Moniz, 1649-028 Lisbon, Portugal; goncalo.seixas@gimm.pt; 7 Faculty of Agriculture, Centre of Excellence One Health Vectors and Climate, Laboratory for Medical and Veterinary Entomology, University of Novi Sad, 21101 Novi Sad, Serbia; mihaela.kavran@polj.edu.rs; 8 Laboratory of Insects & Parasites of Medical Importance, Benaki Phytopathological Institute, 14561 Kifissia, Greece; a.michaelakis@bpi.gr; 9 Ministry of Health, 1148 Nicosia, Cyprus; aoustis@gmail.com (A.S.S.); chantoniou@mphs.moh.gov.cy (C.P.A.); 10 CARE-C, The Cyprus Institute, 2121 Nicosia, Cyprus; af.martinou@gmail.com; 11 Laboratory of Vector Ecology and Applied Entomology, Joint Services Health Unit, British Forces Cyprus, RAF Akrotiri, BFPO57 Akrotiri, Cyprus; 12 Enalia Physis Environmental Research Centre, 2101 Nicosia, Cyprus; 13 MIVEGEC (Maladies Infectieuses et Vecteurs: Écologie, Génétique, Évolution et Contrôle), Université Montpellier, IRD, CNRS, 34394 Montpellier, France; davidroiz@gmail.com; 14 SaBio Group, IREC (UCLM-CSIC-JCCM), 13005 Ciudad Real, Spain; 15 Lokímica Laboratories, 46980 Paterna, Spain; 16 Research and Development Department, Rentokil-Initial Spain, San Fernando de Henares, 28830 Madrid, Spain

**Keywords:** mosquito-borne diseases, mosquito vector, climate change, insecticide resistance, invasive mosquito species, mosquito surveillance, mosquito control, Europe

## Abstract

Mosquito-borne diseases are an emerging public health challenge in Europe, driven by the spread of invasive mosquito species capable of sustaining outbreaks of tropical arboviral diseases. Rising temperatures, shifting precipitation patterns, human-driven habitat changes, and prolonged transmission seasons have increased the risk of dengue, chikungunya, and West Nile virus outbreaks, among other vector-borne diseases. Effective control requires a multifaceted approach, combining traditional and novel methods with advanced surveillance technologies and community involvement. However, growing insecticide resistance and concerns about insecticide use highlight the need for more prudent management of current tools and the development of innovative alternatives. Genetic control strategies, including the Sterile Insect Technique (SIT), *Wolbachia*-based approaches, and genetically modified (GM) mosquitoes, offer promising solutions but still face scientific, regulatory, and societal challenges. This review explores the current landscape of mosquito-borne disease control in Mediterranean Europe, emphasizing key challenges and emerging solutions. An integrated approach that strengthens surveillance, promotes sustainable control methods, and incorporates novel biotechnological tools supported by smart technologies will be essential to reduce the future burden of mosquito-borne diseases in the region.

## Simple Summary

Increasing human mobility and trade, together with land-use and climate changes, are reshaping the Eurasian landscape, making it increasingly suitable for the establishment of invasive vector species and vector-borne pathogens. These transformations are creating an epidemiological scenario that remains largely unpredictable. An additional major challenge is the growing resistance of these vectors to current insecticide control strategies. European countries will need to develop entomological and epidemiological surveillance strategies that are adapted to each region and demonstrate real effectiveness. Biological control approaches, innovative chemical formulations, and genetic strategies such as the sterile insect technique and the use of *Wolbachia*-infected mosquitoes are the new arising strategies. In this new context, integrating emerging technologies with active community participation will be essential to ensure the sustainability and long-term success of vector control policies.

## 1. Current Scenario and Factors Affecting the Spread of Mosquito-Borne Diseases

**Figure 1:**
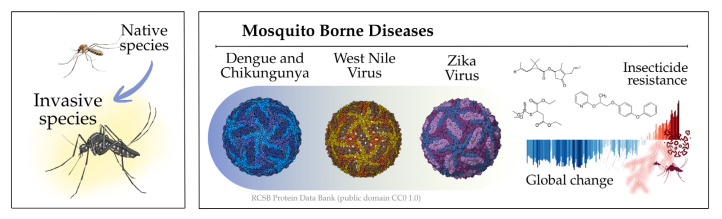
The risks of arboviral outbreaks in Europe are increasing due to the arrival of new invasive vector species supported by global change and insecticide resistance.

Apart from the Arctic, Europe is the world’s fastest-warming area of the world [1], experiencing severe impacts like record heatwaves, increased heat-related deaths, droughts, wildfires, flooding, and threats to food/water security, prompting ambitious EU policies such as carbon neutrality by 2050 and bans on new combustion engine cars by 2035 [2] (even if recently softened), but many climate risks are critical and require urgent action for adaptation and mitigation. Among these climate-related risks, mosquito-borne diseases are an increasing public health concern in Mediterranean Europe, where environmental and climatic changes enhanced the spread of invasive mosquito species and raised the risk of local arboviral transmission (Figure 1). At the same time, the limitations of conventional vector control, particularly insecticide resistance and environmental concerns, highlight the need for more sustainable approaches. This paper reviews the status of mosquito-borne disease risk and control strategies in the region, focusing on key challenges and emerging solutions, including advanced surveillance and novel biological and genetic control methods within an integrated vector management framework.

### 1.1. Relevant Mosquito Vectors in Europe: Native Species and New Arrivals

Mosquitoes are vectors for a variety of pathogens, including viruses and parasites, which can cause diseases such as malaria, dengue, Zika, chikungunya, West Nile fever, dirofilariasis etc. In Europe, the presence of both native and invasive mosquito species has led to increased concerns about the spread of these diseases [3,4] (Table 1).

According to the European Centre for Disease Prevention and Control (ECDC), native mosquito species are those with a distribution range that naturally includes Europe and that can transmit pathogens causing diseases [4]. The most common native mosquito vector species include:

- *Culex pipiens* s.l.: *Cx. pipiens* s.l. is a species complex with several discussed taxa and biotypes that is widespread across Europe. It is a primary vector for West Nile virus (WNV) and can also transmit other pathogens such as Usutu virus (USUV) [5].
- *Culex univitattus*: This is a competent vector for several pathogens, most notably WNV. In Africa and the Middle East, as well as in Portugal, it has been frequently incriminated in association with WNV [6,7,8,9]. The species plays a significant role in the enzootic transmission cycle of WNV, maintaining the virus among bird populations and occasionally transmitting it to humans and other mammals.
- *Anopheles* spp.: Various species of *Anopheles* mosquitoes are found in Europe, including *An. atroparvus* and *An. plumbeus*. These mosquitoes are known vectors for malaria parasites, although the disease is currently not endemic in Europe [10].
- *Aedes* spp.: Native *Aedes* species, such as *Ae. vexans*, are also present in Europe. This species can transmit the nematode *Dirofilaria immitis* and transmit arboviruses like Tahyna, Myxoma, and Rift Valley Fever virus (RVFV) [11,12]. *Ae. vexans* is also possibly competent for WNV [13].

In recent years, several invasive mosquito species of the *Aedes* spp. genus have been established in Europe [4,14]. ECDC defines invasive species as non-native arthropods (like mosquitoes, ticks, sandflies) that are introduced to Europe via trade/travel, establish populations, and can transmit pathogens causing diseases to humans and animals. These *Aedes* species are competent vectors for a number of arboviral diseases and filarial nematodes and pose new challenges for public health.

- *Ae. aegypti*: Commonly known as the yellow fever mosquito, it is a significant vector for several viruses, including yellow fever, dengue, chikungunya, and Zika viruses (respectively, YFV, DENV, CHIKV, and ZIKV). Historically, this species was established in southern Europe but disappeared during the mid-20th century. However, it has recently reappeared in some regions, including Madeira (Portugal), parts of southern Russia, Georgia, the Canary Islands and Cyprus, where it is now established [15]. The re-establishment of *Ae. aegypti* in Europe raises concerns about the potential for autochthonous transmission of the pathogens it carries, especially in southern Europe where climatic conditions are favorable. The species thrives in densely populated areas with inadequate water supply and waste management, making urban environments particularly vulnerable [16,17].
- *Ae. albopictus*: Commonly known as the Asian Tiger mosquito, it is the most invasive mosquito species worldwide including Europe. It has been established in the region since the 1990s and is known as a vector for DENV, CHIKV, and ZIKV [18]. First reported in Europe in 1979 in Albania and later in Italy in 1990, the species is now established in several countries across the EU/EEA, including Austria, Belgium, Bulgaria, Croatia, Cyprus, France, Germany, Greece, Hungary, Italy, Malta, Portugal, Romania, Slovakia, Slovenia, and Spain [19,20].
- *Ae. japonicus*: The Japanese Bush mosquito is another invasive species that has spread across Europe. It has competence for transmitting various arboviruses, including WNV [21]. Since 2020, *Ae. japonicus* has continued to spread across Europe [22]. The species was detected for the first time in southern Poland, where both introduced and already established populations were identified. In addition, further establishment was documented across multiple regions, including northern Czechia, Hungary, northern Italy, the Netherlands, Slovakia, northern Spain, and eastern France. These findings highlight the ongoing spread of *Ae. japonicus* across central and western European regions and underscore the importance of sustained surveillance to monitor its continued expansion [22].
- *Ae. koreicus*: The Korean bush mosquito is a relatively recent invasive species in Europe and has demonstrated laboratory competence for transmitting *D. immitis* and chikungunya virus (CHIKV) under specific experimental conditions [23]. Since 2020, surveillance data indicate that the species has continued to expand its distribution showing further spread in Hungary and Switzerland [24]. Earlier records confirm that the species is already established in several European countries, including Belgium, where it was first detected in 2008, Italy, Slovenia, and others. Continued monitoring remains essential to assess its public health relevance and to track its evolving European range.

Regarding the implications for disease transmission, the co-existence of both native and invasive mosquito species substantially increases the risk of arboviral transmission. Invasive species, in particular, have been directly associated with several mosquito-borne disease outbreaks in Europe [25]. *Ae. albopictus* has been linked to outbreaks of chikungunya and dengue in southern European countries, underscoring its growing public health relevance [26,27]. As these invasive vectors become established and expand their geographic range, the likelihood of local transmission of pathogens previously considered exotic increases, thereby elevating the overall epidemiological risk across the region.

**Table 1:** Main native and invasive species of mosquitoes capable of transmitting pathogens that can be currently found in Europe.

Genus	Species	Vectorial Capacity	Distribution
*Culex*	*Cx. pipiens* s.l.	WNV, USUV, RVFV transmission as the main problem [28,29,30]; vectorial capacity for JEV just in laboratory conditions [31]; SINV, TAHV present in natural infections [32]; dirofilarial worms [33], avian malaria [34].	Native to most of urban, peri-urban and rural areas of Europe [35,36].
	*Cx. univitattus*/ *Cx. perexiguus*	WNV, RVFV and SINV (originally isolated in this species) were reported linked to this vector [9,37,38]. These species play a significant role in the enzootic transmission cycle of WNV [38].	Mainly located in North Africa and southern parts of Europe (Morocco, Algeria, and some areas of the Iberian Peninsula). Absent from most of Europe except parts of the eastern Mediterranean, like Turkey [39].
	*Cx. theileri*	Vector competence for WNV at laboratory conditions [40], and *D. immitis* [41].	Present in southwestern Europe, northern Africa, and parts of the Middle East. Isolated occurrences in eastern Europe, absent at most of central and northern Europe [42].
*Aedes*	*Ae. aegypti*	Principal vector of YFV, DENV, CHIKV and ZIKV [43], competence for MAYV observed in laboratory conditions [44].	Historically established across the Mediterranean region, the Caucasus and the Atlantic archipelagos. In Europe its current distribution is limited but expanding [22,42].
	*Ae. albopictus*	Competent for CHIKV, DENV, ZIKA, dirofilariasis, and other 22 arboviruses including YFV, RVFV, JEV, SINDV, LACV, OROV, USUV and MAYV [45].	It is native to Asia and is now widely established across southern and central Europe, reaching Belgium and central Germany to the north [22,42].
	*Ae. japonicus*	Several studies have shown competence in WNV [46,47], JEV, LACV [48] and Eastern equine encephalitis virus, CHIKV, DENV [49], and RVFV [50,51,52].	It appeared in northern France in 2000 with subsequent introductions in Belgium, Switzerland, Germany, Czech Republic, Austria, Slovenia, northern Italy, Croatia [22,42].
	*Ae. korei* *c* *us*	It is suspected to be a vector for the JEV [53]. It has been linked also to *Brugia malayi* and *D. immitis* in dogs [54].	First reported in Belgium in 2008, established in Germany, Hungary, the Netherlands, Austria, Switzerland, northern Italy, Crimean peninsula and Slovenia [22,42].
	*Ae. cretinus*	Evidence for vector competence is very limited and, for arboviruses, absent.	This species is native to the Eastern Mediterranean basin and Black Sea region, like Turkey, Greece or North Macedonia [55,56,57].
	*Ae. vexans*	It can transmit TAHV and *D. immitis* [58] and it is a potential vector of WNV [59] and RVFV [60].	It is widely distributed across Europe, particularly in Central Europe, Occidental Russia, and the Mediterranean basin [22,42].

WNV = West Nile virus; USUV = Usutu virus; RVFV = Rift Valley Fever virus; JEV = Japanese Encephalitis virus; SINV = Sindbis virus; TAHV = Tahyna virus; YFV = Yellow Fever virus; DENV = Dengue virus; CHIKV = Chikungunya virus; ZIKV = Zika virus; MAYV = Mayaro virus; LACV = La Crosse virus; OROV = Oropouche virus.

### 1.2. Mosquito-Borne Diseases: Recent Outbreaks in Europe and Potential Risks in the near Future

#### 1.2.1. *Aedes*-Borne Arboviruses: Dengue and Chikungunya Virus

*Ae. albopictus* and *Ae. aegypti* are the primary vectors of DENV and CHIKV. Their recent introduction to Europe and rapid expansion, particularly since the early 2000s, have redefined the emergence risk of these two arboviruses in newly colonized territories (see above).

Dengue is an endemic and epidemic disease in tropical and subtropical regions. The DENV1 serotype was first isolated in 1943 by Hotta and Kimura, and other serotypes were isolated between 1944 and 1957. Humans are the primary reservoir for this flavivirus (*Orthoflavivirus* sp.), with infection caused by one of the four serotypes (DENV1-4). The consequences can be lethal, especially in children under 5 years of age. The reasons for the severity of cases remain poorly understood and are multifactorial [61]. Dengue virus circulation has exploded since the mid-20th century, with an estimated 50 million annual infections [62]. Nearly half of the world’s population currently lives in areas at risk of dengue transmission.

CHIKV, a togavirus (*Alphavirus* sp.), causes high fever and severe joint pain, which can last up to three weeks. While relatively low in pathogenicity, it can lead to sequelae lasting several months [63]. Major outbreaks have been observed since the early 2000s in South America, the Caribbean, Africa, and Asia, increasing the risk of importation by travelers into Europe and North America.

DENV and CHIKV are not endemic to Europe but are regularly introduced by viremic travelers returning from outside continental Europe. In regions colonized by *Ae. aegypti* and *Ae. albopictus*, factors such as travel volume and the intensity of DENV and CHIKV circulation in travelers’ regions of origin are the primary risk variables for the emergence of autochthonous outbreaks in European countries [64].

Despite initial doubts about the environmental and climatic suitability of European countries for these diseases, the first autochthonous outbreaks of chikungunya in Italy in 2007 (330 cases), followed by chikungunya (2 cases) and dengue (2 cases) in France in 2010, as well as in Croatia (10 cases), confirmed the possibility of local transmission in temperate zones. Since the first Italian outbreak, *Ae. albopictus* has been the responsible vector for 2033 cases of chikungunya and 605 cases of dengue in Europe [65,66]. Italy and France account for 98.5% of these cases (2602). In 2025, the importation of numerous chikungunya cases, followed by the establishment of local transmission chains and the movement of viremic cases led to the multiplication of outbreaks in Italy and France marking the largest European epidemic to date (1172 cases, with 384 in Italy and 788 in France). Although the number of imported chikungunya cases in 2025 is comparable to previous years’ imported dengue cases, *Ae. albopictus*’s competence for CHIKV, particularly the ECSA (East-Central-South Africa) strain [67], has contributed to the rapid infection of vectors and the unprecedented increase in infection cases in Europe.

Among the recognized risk factors, local vector population density, warm summer temperatures, tourism, and the movement of travelers from endemic areas—particularly from French overseas territories—are key determinants for the appearance of European outbreaks [64,68]. Areas with medium population density, peri-urban zones, and significant vegetation are the most favorable environments for vector circulation [69]. Increasing travel flow within Europe in recent years raises concerns about potential viral inoculum from intra-European movements, making the occurrence of outbreaks less predictable. Furthermore, global climate change, particularly the rising temperatures in Western Europe, is already contributing to the expansion of receptive territories and transmission periods compatible with local transmission. This observable trend is expected to increase over the next decades [68].

#### 1.2.2. Zika Virus

While *Ae. albopictus* has shown limited efficiency in Zika virus (ZIKV, Flaviviridae; Orthoflavivirus) transmission, the disease remains a concern in tropical regions and has been implicated in several major outbreaks in the Americas. Although vector-borne transmission of Zika is possible in Europe (in France 2019 [70]) at moderate levels of introduction it presents only a low epidemic potential compared to dengue and chikungunya [71].

#### 1.2.3. West Nile Virus

WNV is a flavivirus (*Orthoflavivirus* sp.) that is relatively low in pathogenicity, often asymptomatic but capable of causing meningitis and potentially fatal encephalitis. The transmission cycle involves avian reservoirs (particularly corvids), including migratory species [72]. Potential native vector species belong primarily to the genus *Culex*, with *Cx. perexiguus* and *Cx. modestus* that are recognized as vectors in southern Europe, particularly in Spain and the Mediterranean basin. *Cx. pipiens* s.l., is a ubiquitous mosquito species with ornithophilic biotypes (*Cx. pipiens* biotype *pipiens*) that is found everywhere throughout Europe, and is the major WNV vector, responsible mainly for enzootic avian cycles, but also capable of infecting mammalian hosts, for example horses [73].

Between 2015 and the end of 2025, more than 8000 human infections have been recorded, with meningitic forms responsible for nearly 600 deaths [74]. Approximately 80% of cases are asymptomatic, indicating that many thousands of individuals are likely infected each year. While the overall risk is considered low, the exposure of the European population is considerable. As a result, WNV has become the deadliest arbovirus in Europe. In addition to annual deaths (up to 166 in 2018), the virus necessitates costly blood and organ exclusion or control measures in affected countries [75]. These measures limit blood availability for medical needs, creating significant economic and healthcare impacts.

Southern and Eastern Europe are the most favorable regions for WNV transmission (Greece, Italy, Romania). However, since 2020, WNV has gradually expanded its circulation further north (Austria, Germany [76], Netherlands, Belgium). In 2025, 1096 locally acquired human cases of WNV infection with known place of infection were reported in Europe, including Italy (with 773 cases), Greece, Serbia, France, Romania, Spain, Hungary, Croatia, Albania, Germany, North Macedonia, Bulgaria, Kosovo, and Türkiye. Among these reported cases, 95 resulted in fatalities [77,78].

The role of birds as viral reservoirs plays a major part in the virus’s dissemination along migratory corridors. Limited knowledge of migratory routes, their annual variations, and the impacts of climate change, along with the potential for WNV to infect a wide range of bird species with varying susceptibility to infection, creates diverse transmission cycles that vary by region and make the emergence of outbreaks unpredictable. The diversity of *Culex* mosquito species involved in outbreaks, along with their trophic preferences, local dynamics, and the variety of potential avian reservoirs, further complicate the epidemic cycles in Europe.

Six WNV lineages circulate in Europe, with varying pathogenicities [79]. Urbanization and migratory bird movements across Europe alter the historical distribution of these lineages. Climate change, including hot and dry summers—aggregating bird and vector species around water sources—and mild winters, which extend the activity period of vectors and the survival of overwintering individuals, favors the overall ecological suitability of Europe for WNV [80].

### 1.3. Global Change and the Spread of Arboviral Diseases

Climate change is determining significant shifts in the distribution of plants and animals as a response to changing habitats. Mosquitoes are also involved in this process and along with the mosquitoes come arboviral diseases [81]. An example of this phenomenon is the rise in dengue cases in the Mediterranean region, which was previously only sporadically affected by this disease [82]. This situation is worsened by globalization and population mobility, which facilitates the movement of vectors and pathogens that increase their chance to expand their geographic range [83]. The Intergovernmental Panel on Climate Change identifies the population dynamics of vectors as one of the events most likely to be impacted by global warming [84].

Not all the climate variables being altered will affect mosquito species in the same way. Changes in rainfall patterns, the rise in global average temperatures, and extreme weather events such as floods or erratic rainfall will directly impact the vectors. Other factors, such as rising sea levels, flooding of coastal areas, disruption of natural environments, species loss, droughts, and changes in natural phenomena like ocean currents, may indirectly affect the vectors if they create new suitable habitats. All these factors can lead to an increase and expansion of vector-borne diseases. In fact, there is already evidence of a link between the anomalies observed in the El Niño phenomenon and disease outbreaks, due to the underlying droughts and flooding [85,86].

Temperature is one of the most important abiotic factors affecting the risk of arboviral disease outbreaks. As an example, temperatures above 35°C may disrupt the transmission of certain pathogens and reduce vector activity, while temperatures around 30 °C could be optimal for vector transmission [87]. Within certain limits, a higher water temperature shortens the life cycle of mosquitoes [88], reducing the developmental time of the different stages. Also, it enhances the digestibility of blood in females [89], leading to an increase in feeding frequency and, consequently, to a greater probability of pathogen transmission if the female is infected. Furthermore, in temperate climate regions such as Europe, warmer winters correspond to an increased length of period suitable for mosquito activity. Evidence from Greece shows that WNV circulation can persist during winter months, with WNV-positive *Cx. pipiens* s.l. detected in multiple sites during winter 2022, marking the first documentation of off-season circulation in adult mosquito populations in temperate Europe [90]. This suggests that milder winters and altered climatic patterns may enable extended or year-round virus activity. Additionally, the recent detection of Usutu virus (USUV) in *Culex* spp. in Northern Greece underscores the ongoing emergence and establishment of novel or previously neglected arboviruses within Europe’s changing ecological landscape [91]. Together, these findings illustrate how global change, through urban expansion, climate-driven shifts in vector ecology, and the introduction or persistence of new pathogens, contributes to the increasing unpredictability and geographical expansion of arboviral threats.

Flooding can lead to an increase in the number of breeding habitats, thereby boosting the population [92]. These conditions favor higher vector survival and reproduction, potentially leading to increased vector abundance and an elevated risk of zoonotic and vector-borne disease transmission [93].

Certain mosquito species, *Ae. albopictus* among others, are characterized by their rapid adaptation to climatic change at levels higher than expected [94].

Urbanization is also playing a critical role in reshaping the epidemiology of arboviral diseases worldwide. A recent systematic review following PRISMA guidelines demonstrated that urbanization is strongly associated with increased *Aedes* mosquito densities and the amplification of arboviral transmission [95]. Across 29 studies analyzed, variables such as human population density, urban growth, and changes in artificial landscapes consistently correlated with higher vector abundance, with densities exceeding 1000 inhabitants/km^2^ linked to elevated arboviral disease levels. These findings highlight how expanding urban environments create favorable ecological niches that enhance both *Aedes* ecology and transmission dynamics. At the same time, global change also affects the behavior and seasonality of other mosquito vectors [95].

### 1.4. Insecticide Resistance in Europe

Insecticide resistance has become one of the most pressing challenges for mosquito control programs in Europe. The extensive and recurrent application of chemical insecticides, particularly pyrethroids, in both public health and agricultural settings has imposed strong selection pressure on mosquito populations. This has accelerated the evolution of resistance mechanisms across multiple mosquito species of epidemiological relevance, including *Ae. aegypti*, *Ae. albopictus*, and *Cx. pipiens* s.l. The establishment and rapid spread of invasive mosquito species in temperate European regions further exacerbates this issue, with resistance patterns increasingly resembling those of tropical zones [96,97].

Mechanistically, insecticide resistance arises through multiple adaptations, including target-site mutations, enhanced metabolic detoxification, cuticular changes, and behavioral avoidance. Among the most well-characterized mechanisms are knockdown resistance (*kdr*) mutations in the voltage-gated sodium channel (*VGSC*) gene, which reduce the efficacy of pyrethroids and DDT. In *Ae. albopictus*, the *V1016G*, *F1534C*, and *I1532T* mutations are now widely reported in European populations, notably in Italy, Greece, and Spain [98,99,100]. Functional validation assays by Kasai et al. [101] confirmed that the *V1016G* mutation confers significantly higher resistance levels than *F1534C* or *F1534S*, raising concerns about the diminishing effectiveness of commonly used adulticides.

In *Ae. aegypti*, resistance has been extensively studied in Madeira Island, Portugal. Bioassays conducted in 2013 demonstrated high levels of resistance to multiple insecticide classes, with mortality ranging from 10% to 78% for permethrin, cyfluthrin, and fenitrothion [102]. Molecular analyses confirmed the near fixation of the *F1534C* mutation and moderate frequencies of *V1016I*, along with elevated activity of detoxification enzymes (cytochrome P450s, esterases, and GSTs). Together, these mechanisms have rendered *Ae. aegypti* from Madeira resistant to pyrethroids, organophosphates, and carbamates, thus severely limiting chemical intervention options.

In *Cx. pipiens* s.l., resistance is widespread and genetically diverse. The *G119S* mutation in the acetylcholinesterase gene (ace-1), associated with resistance to organophosphates and carbamates, is prevalent in Italy and Greece, often exceeding 50% allele frequency [103,104]. Pyrethroid resistance is increasingly mediated by the *L1014F kdr* mutation, now reported across much of Europe, including Hungary, where its frequency reaches 36% [105]. Studies from Belgium also report widespread phenotypic resistance to permethrin and deltamethrin, as well as reduced susceptibility to bendiocarb and *Bacillus thuringiensis israelensis* (Bti) [106]. In Germany, early signs of pyrethroid tolerance have been documented in *Cx. pipiens* s.l. and *Cx. pipiens molestus*, suggesting a continued northward expansion of resistance [107].

Another critical concern is resistance to diflubenzuron (DFB), a chitin synthesis inhibitor widely used in European mosquito control. In Northern Italy, *Cx. pipiens* s.l. populations were found to harbor *I1043M* and *I1043L* mutations in the chitin synthase gene (chs-1), associated with up to 2900-fold resistance to DFB [104]. More recently, the *I1043F* mutation was detected in Crete [108], confirming the geographic expansion of DFB resistance in Europe. These findings threaten the long-term viability of DFB, one of the few larvicides still approved for use under European biocide regulations.

Regarding *Ae. albopictus*, knockdown resistance mutations conferring resistance to pyrethroids were found to be present in several populations both in the west and in the east of Mediterranean Europe [109].

## 2. Surveillance and Control of Mosquito-Borne Diseases in Mediterranean Europe

### 2.1. Entomological and Epidemiological Surveillance of Mosquitoes and Mosquito-Borne Pathogens

**Figure 2:**
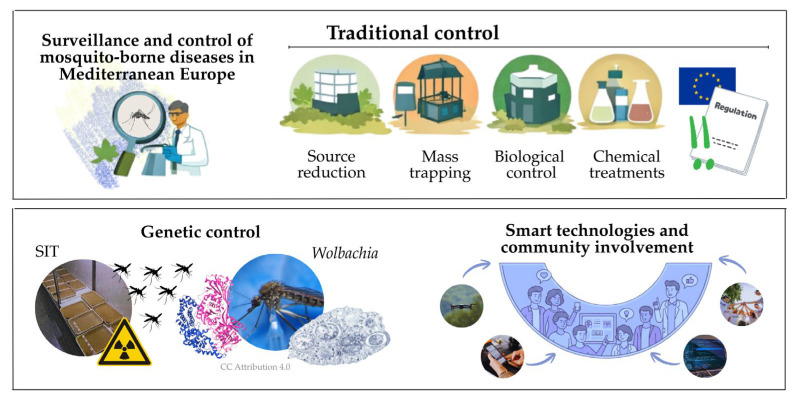
Surveillance and control measures applied in Europe and the possible contribution by innovative control strategies and community involvement.

When discussing surveillance (Figure 2), it is very important to differentiate between surveillance and monitoring. Mosquito or pathogen monitoring includes detection or quantification of these organisms in a specific area determined by sampling, with no further action required afterward. In contrast, surveillance obliges suppression of vectors and pathogens aiming to reduce the hazard or minimize the likelihood for disease outbreak.

From medical entomology and the public health point of view, there are two major types of surveillance:

1. Entomological surveillance: aims to detect and examine the population of the invasive and native mosquito species, which are potentially harmful to human and animal health as proven vectors.
2. Epidemiological surveillance: focuses on existing or threatening outbreaks caused by mosquito-borne pathogens.

The general goals of surveillance can be classified as: 1. Early detection: identify new introductions of invasive mosquito species in previously unaffected areas; 2. Population monitoring: track changes in population density and distribution over time; 3. Risk assessment: evaluate the potential for disease transmission to occur in a region; and 4. Efficacy evaluation: assess the success of control interventions, like source reduction and insecticide application.

A surveillance program for mosquito-borne diseases must be tailored to each territory based on the likelihood of viral circulation. Several considerations must be taken into account when designing such a program, as exemplified by WNV surveillance:

- Vectors of pathogens: the primary vectors are mosquitoes of the genus *Culex*. Therefore, the surveillance program should prioritize areas with favorable climatic and environmental conditions for their breeding and survival.
- Epidemiological reservoirs: migratory birds serve as the main epidemiological reservoirs, playing a key role in the dissemination of the virus across different geographic regions.
- Risk areas: wetlands, such as river deltas, marshy areas, or lakes which host abundant migratory birds and mosquitoes, are optimal habitats for the spread of the disease and should be closely monitored.
- Sentinel species: equines play a prominent role as sentinels, under certain circumstances, since they are more exposed to the bites of the vector transmitting the virus than humans [110].

The selection of tools and methods for surveillance is heavily influenced by the biology of the targeted mosquito and the pathogen. This necessitates a comprehensive understanding of all stages of the mosquito life cycle and all aspects of the pathogen’s transmission cycle. These factors must be strategically considered when planning surveillance activities [111,112,113].

Both entomological and epidemiological surveillance can be conducted actively or passively. For example, active surveillance involves direct methods such as mosquito sampling, while passive surveillance relies on data collected indirectly (i.e., by citizen science applications).

Out of all aforementioned invasive mosquito vectors, the most dangerous one in Europe is *Ae. albopictus* and many European countries including Spain, Italy, and Germany invest significant efforts in monitoring this species [114,115,116]. The second most threatening invasive species is *Ae. aegypti* due to its vectorial competence to transmit severe pathogens. The third phase belongs to *Ae. japonicus*, which is more widespread than *Ae. aegypti* but with a reduced health-related significance.

Mosquito surveillance may target various stages of the mosquito life cycle, including eggs, immature stages (larvae and pupae), and adults. Depending on the species, different traps are used. *Ae. albopictus* is primarily monitored/surveyed by ovitraps. Surveillance in the immature stages involves inspecting potential mosquito breeding sites, i.e., water-holding recipients, both natural and artificial (tires, birdbaths, flowerpots) in residential and public areas which helps to identify breeding hotspots.

Adult mosquito traps vary, and include BG-Sentinel traps, EVS traps, CDC Light traps, and Gravid Traps [4]. All adult traps share the common goal of attracting and capturing adult mosquitoes.

Epidemiological surveillance differs significantly from the surveillance of invasive mosquito species due to the complexity of pathogen biology. Regardless of the epidemic being monitored, the first step is to identify not only all factors affected by disease, but also those involved in its transmission and dissemination. In some cases, the transmission chain is simpler, making surveillance less complicated. For example, dengue surveillance compared to WNV surveillance.

Dengue surveillance involves systematic collection, analysis, and interpretation of data related to dengue virus infections in humans and vectors. Dengue is caused by four virus serotypes DENV-1 to DENV-4 and is primarily transmitted by *Ae. aegypti* and *Ae. albopictus*. Surveillance includes tracking human cases to detect outbreaks and assess disease burden, as well as monitoring mosquito vectors to assess the risk of transmission.

Unlike dengue, pathogens with complex biology, such as WNV, make tracking and surveillance considerably more challenging. This virus primarily affects birds, which serve as its primary hosts and reservoirs, including both domestic and wild birds, while humans and horses act as incidental dead-end hosts.

In several countries, recurrent transmission to humans and equines highlights the need for sustained surveillance efforts. The 2010 WNV outbreak in Greece further illustrated these complexities: despite substantial public health and socio-economic impacts, the epidemic was brought under control only after major investments in prevention and response. Subsequent cost–benefit analyses showed that illness and prevention costs declined notably in the years following the outbreak, with households expressing a willingness to pay 22–27 € annually to eliminate the mosquito problem [117]. These findings underscore that, while coordinated interventions can generate a net socio-economic benefit, the potential spread and wider consequences of WNV in the absence of control measures remain unpredictable and difficult to quantify.

Thus, entomological surveillance is critical for this pathogen, because the major vector mosquito species is the European native *Cx. pipiens* s.l. Therefore, surveillance in endemic countries should include: 1. Detection of the virus in mosquitoes: monitoring mosquitoes as vectors; 2. Bird surveillance: tracking viruses in birds, which are the main source of infection; 3. Sentinel animals: using sentinel animals, such as chickens, for early detection [118]; 4. Reservoirs and dead-end hosts: detecting the virus in humans and horses, which act as dead-end hosts [118].

In the following text, some examples of the good practices of epidemiological surveillance in Europe are described.

#### 2.1.1. Italy, WNV Surveillance

In Italy, WNV surveillance is regulated by the National Prevention, Surveillance, and Response Plan for Arboviral Diseases (PNA) 2020–2025, approved in January 2020. The plan mandates integrated human, animal, and entomological surveillance, recognizing the importance of veterinary data in assessing human risk. It also includes monitoring for USUV in areas where both viruses circulate, due to their similar transmission cycles.

Human surveillance is conducted nationwide, with intensified efforts from May to November in endemic regions. Diagnosis of WNV and USUV is prioritized in these areas, and cases in blood, organ, and tissue donors are closely monitored to implement preventive measures. Surveillance is coordinated nationally by the Istituto Superiore di Sanità and the Ministry of Health, which report data to the European Commission and ECDC. Regional authorities manage their own surveillance protocols and share findings with national authorities. Results are regularly published in bulletins by the ISS, in collaboration with the Ministry of Health and IZS Teramo [119].

#### 2.1.2. Serbia, WNV Surveillance

The integrated WNV monitoring program, established by the Veterinary Directorate of the Ministry of Agriculture, has been active since May 2014 and is ongoing. The primary aim of the program is the early detection of WNV presence in the environment to enable timely control measures, including vector control and the prevention of disease outbreaks in humans and animals.

The surveillance program is based on monitoring antibodies in sentinel animals (WNV IgG in horses and poultry in 2014; WNV IgM antibodies in horses from 2015 to 2021; and from 2022, IgM antibodies in horses and IgG antibodies in calves), as well as monitoring the presence of the virus in natural hosts and vectors, wild birds and mosquitoes. The One-Health approach, implemented by a multisectoral team in Vojvodina Province, has been crucial for WNV surveillance.

Animal sampling is conducted by the Scientific Veterinary Institute “Novi Sad,” mosquito sampling by the Laboratory of Medical and Veterinary Entomology/Center of Excellence—One Health, and human population surveillance by the Institute of Public Health of Vojvodina [73,118]. Mosquito sampling in Vojvodina Province, partly supported by the Provincial Government of Vojvodina—the Secretariat of Urbanism and Environmental Protection, focuses on analyzing the main vector, *Cx. pipiens* s.l. Mosquitoes are collected at 65 locations once or twice a month from May to October.

When a positive sample is detected, final confirmation is required from the reference laboratory. Subsequently, authorities are informed to guide further activities in epidemic prevention and suppression.

#### 2.1.3. Spain, WNV Surveillance

The Ministry of Agriculture, Fisheries, and Food of Spain established the WNV surveillance program. The results of the surveillance plan aim to determine the presence or absence of viral circulation. If viral circulation is detected, the plan provides the basis for an appropriate and effective response through the adoption of preventive measures aimed at mitigating the risk to animal and public health posed by the spread of this disease.

Considering the aforementioned points, the objectives of the surveillance plan are: 1. Detection of viral circulation: identify risk areas where the disease can spread and cause outbreaks; 2. Assess the risk of disease emergence from the perspective of animal and public health to provide a timely and effective response; 3. Evaluate the need to implement specific control measures and schedule them appropriately [13].

Seasonal surveillance plan: Given the seasonal nature of the disease, the implementation dates are aligned with the mosquito activity period. Therefore, the program starts between March and April, depending on entomological data, and ends in January of the following year.

Plan Implementation Areas: Humid areas such as river deltas, marshy regions, and lakes with an abundance of migratory birds and mosquitoes are optimal habitats for the spread of WNV. Considering the history of WNV in neighboring countries and the data obtained from surveillance in Spain, areas of varying risk levels will be defined based on specific criteria.

The plan includes testing sentinel birds, wild birds, mosquitoes, and surveillance of horses [110].

#### 2.1.4. France, DENV, CHIKV and WNV Surveillance

Due to the presence of the *Ae. albopictus* vector in metropolitan France, dengue, chikungunya, and Zika have been monitored by a surveillance program since 2006. Throughout the year, these arboviruses are subject to mandatory reporting, requiring health professionals to report all biologically documented cases to the Regional Health Agencies (ARS: Agence régionale de santé). The surveillance system is reinforced annually from May to November, during the peak activity period of *Ae. albopictus*.

Awareness campaigns are launched at the beginning of the season to inform health professionals, doctors, and laboratories about the risk of arbovirus transmission and the importance of case reporting. Additionally, an automated system for transferring results from the Eurofins Biomnis and Cerba laboratories helps identify unreported cases. Each identified case triggers an epidemiological investigation by the ARS and prompt intervention by vector control services (LAV: services de lutte antivectorielle) around potentially viremic cases to prevent local transmission of the virus.

A case is considered autochthonous when a person who has not traveled for 15 days before the onset of clinical signs is infected by a local mosquito, which itself became infected after biting a viremic person returning from an endemic area. The National Reference Center (CNR: Centre national de référence) for arboviruses is responsible for the confirmation of the first autochthonous cases during local transmission events.

When an autochthonous case is identified, an active search for additional cases is immediately initiated in the surrounding area. This includes door-to-door surveys within a 150–250 m radius, communications with health professionals, and a press release to raise awareness among the general population. Every situation of autochthonous transmission undergoes a risk assessment regarding the safety of health products of human origin [111].

Since 2021, human surveillance of WNV infection has been based on mandatory notification of biologically documented cases—whether imported or autochthonous—classified as confirmed or probable. Previously, surveillance relied exclusively on data from the National Reference Center (NRC) for arboviruses, which for many years was the only laboratory performing diagnostic testing. Currently, WNV diagnosis is also conducted by a limited number of hospital-based and private laboratories.

In parallel, animal surveillance systems are in place. Neuroinvasive WNV disease is a notifiable condition in equids, and a syndromic surveillance system is implemented through a national network of veterinarians (RESPE). Surveillance in birds is carried out by the Animal Health Laboratory (ANSES/National Reference Laboratory) in collaboration with the French Office for Biodiversity (OFB) [120]. Operational research studies are currently underway to assess the added value of monitoring WNV circulation in *Culex* mosquito vectors within the French epidemiological context.

Overall, WNV epidemiological surveillance in France has, for many years, followed a One Health approach. It relies on close collaboration between Santé publique France, the NRC for arboviruses, ANSES/National Reference Laboratory, the OFB, research teams, and mosquito control operators. The primary objective of this integrated surveillance system is the early detection of WNV circulation, in particular to ensure the safety of substances of human origin and to prevent severe clinical outcomes.

#### 2.1.5. Croatia, DENV Surveillance

The Croatian Institute of Public Health (CIPH) coordinates with county public health institutes to manage the epidemiological situation within their respective counties. Following directives from the national institute, county institutes develop programs to address potential outbreaks. The reporting of communicable disease outbreaks in Croatia is regulated by several laws and ordinances, including the Act on the Protection of the Population against Communicable Diseases (OG 79/2007; 113/2008; 44/2009; 130/2017; 114/2018; 47/20; 134/20; 143/21), the List of Communicable Diseases (OG 60/14; 28/20; 73/22), and the Ordinance on the Method of Reporting Communicable Diseases (OG 9/24).

According to these regulations, each communicable disease outbreak must be immediately reported to the Infectious Disease Epidemiology Division of the CIPH upon identification. The division receives a detailed paper report after the outbreak investigation is completed.

Outbreaks are investigated by field epidemiology teams, supported by one of the 21 county public health laboratories. These teams also collaborate with state sanitary inspectors, enabling environmental analysis and sample collection for laboratory investigation. In some cases, veterinary inspection is involved, allowing for the collection of food samples of animal origin.

Reporting covers outbreaks of all infectious diseases listed in the List of Communicable Diseases of interest to Croatia. The CIPH is also mandated to investigate outbreaks of unknown origin, covering the entire range of microbiological agents and outbreaks caused by toxins.

The Strengthening and Upgrading Croatia’s Communicable Disease Surveillance System—SUCCESS project aims to improve the monitoring and surveillance of communicable diseases in Croatia. This project focuses on the informatization and digitalization of the health system to ensure higher quality data, simpler and faster availability, and better analytics for assessing the epidemiological situation, while reducing the burden on healthcare professionals. The goal is to enhance the monitoring, assessment, and planning of interventions and responses to communicable disease threats by upgrading the existing national IT system for surveillance (NAJS). This initiative aligns with Regulation (EU) 2022/2371 of the European Parliament and of the Council on serious cross-border threats to health, repealing Decision No. 1082/2013/EU.

#### 2.1.6. Portugal, WNV and DENV Surveillance

Portugal has implemented a comprehensive national vector surveillance program under the National Vector Surveillance Program—REVIVE (REde de VIgilância de VEctores). Operational since 2008 and overseen by the Ministry of Health, REVIVE monitors mosquito populations across the country and provides essential data for managing vector-borne disease risk, including surveillance of WNV and *Aedes*-borne viruses [121,122]. Complementing REVIVE, the Directorate-General of Health (DGS) has developed the National Plan for the Prevention and Control of Vector-Borne Diseases, which integrates entomological and epidemiological approaches into national prevention, surveillance, and control strategies.

West Nile virus surveillance is a key component of Portugal’s public health preparedness [123]. REVIVE conducts regular monitoring of mosquito populations, particularly *Culex* species, the main vectors of WNV, through systematic breeding-site inspections and routine mosquito trapping for laboratory testing to detect the presence of the virus. Surveillance is further strengthened by monitoring sentinel animals, including birds and horses, which can provide early warning of viral circulation in the environment [124,125].

Although WNV cases in Portugal remain sporadic, prevention efforts continue to focus on early detection and avoidance of larger outbreaks. Public health campaigns encourage protective behaviors such as the use of repellents, appropriate clothing, and eliminating standing water—breeding sites. Ongoing research and continuous refinement of surveillance methodologies aim to improve the country’s capacity to detect and respond to WNV circulation [126].

Dengue surveillance in Portugal is a central component of the national public health action against vector-borne diseases, given the presence of both *Ae. aegypti* and *Ae. albopictus* species. REVIVE conducts regular monitoring of *Aedes* populations, including mapping of breeding sites, mosquito trapping for laboratory analysis and environmental assessments of factors that may favor mosquito proliferation [127,128,129]. Human dengue surveillance, particularly during the summer mosquito season, focuses on tracking both imported and locally acquired cases to identify potential transmission events and enable rapid intervention. Public health campaigns promote personal protection and environmental management, reinforcing community involvement in preventing dengue transmission [130].

In addition to national efforts, Portugal collaborates closely with international agencies, including the World Health Organization (WHO) and ECDC. These partnerships enhance a country’s surveillance capacity by providing access to global datasets, methodological guidance, and best practices for vector-borne disease prevention and control [131].

#### 2.1.7. Greece, Surveillance of WNV and *Aedes*-Borne Diseases Imported Cases

In Greece, the prevention of WNV transmission is addressed through a coordinated national strategy that combines three critical components: comprehensive disease surveillance, systematic mosquito population control, and public education on bite prevention measures. The Hellenic National Public Health Organization (NPHO) maintains this integrated approach through continuous monitoring and seasonal response protocols. Since 2010, Greek health authorities have activated enhanced surveillance protocols annually from June to November, coinciding with peak mosquito activity [132,133]. The NPHO works in partnership with regional health directorates, municipal authorities, and specialized laboratories to implement these measures. The Greek surveillance system prioritizes early human case detection through multiple coordinated mechanisms. Before each transmission season, the NPHO distributes updated diagnostic protocols to healthcare facilities across the country, establishing mandatory testing requirements for patients presenting with acute neurological symptoms including meningoencephalitis or flaccid paralysis, as well as those with unexplained febrile illness in endemic regions. A centralized laboratory network, coordinated by the Vector-borne Diseases Unit of NPHO, provides standardized diagnostic testing at no cost to patients while maintaining real-time electronic reporting to the national surveillance system. Complementary blood safety measures are implemented through the national transfusion service, which conducts targeted screening in affected areas with immediate reporting of positive results to the NPHO [134]. When cases are confirmed, rapid notification protocols activate local health units while weekly epidemiological reports guide response strategies. This triggers regional and municipal authorities to implement targeted vector control interventions in identified risk areas. This multi-sectoral approach demonstrates Greece’s commitment to meeting international health standards while adapting measures to local epidemiological and environmental conditions [135]. The system’s effectiveness relies on the sustained cooperation between clinical, laboratory, and public health professionals across all administrative levels.

Since 2016, Greece has maintained a structured national system for managing imported cases of DENV, ZIKV, and CHIKV, formalized through a ministerial circular of the Ministry of Health. Developed with technical support from the EU co-funded LIFE CONOPS project, the framework provides concise core guidance supported by detailed technical instructions for field implementation (see Annex 7: Standard Operational Procedures for quality control on emergency treatments [136]). It clearly outlines the responsibilities of national, regional, and local authorities, offering standardized procedures for surveillance, risk assessment, and vector control. This tiered and operationally focused approach enables rapid coordination and effective response, mirroring the overall logic applied in Greece’s WNV management system.

#### 2.1.8. Cyprus, WNV Surveillance and Management of *Aedes* Invasive Species

The Republic’s mosquito management is led by the Ministry of Health (MoH) and coordinated by the Public Health Services (PHS), supported by the multi-stakeholder Committee for the Prevention and Management of Tropical Diseases (CPMT).

Historically, vector control focused on malaria vectors (*Anopheles* species) as well as *Culex* species—the primary WNV vectors—using larval source management in rural and urban water bodies including the use of *Bti*. Surveillance for WNV was significantly intensified following a 2019 outbreak involving 23 confirmed cases, 15 of which presented with the neuroinvasive form of the disease. Current state measures include routine blood donor screening during the summer and early autumn months, while the Veterinary Services maintain a national monitoring system for WNV detection in bird and horse populations.

Regarding the *Aedes* invasive species, following the detection of *Ae. aegypti* in 2021 and *Ae. albopictus* in 2022 [15], the MoH established a fully operational high-density network for surveillance, consisting of standardized ovitraps and BG-Sentinel traps at strategic locations. Vector control measures are based on extensive daily Door-to-Door (DtD) source reduction. Field teams utilize GIS-integrated real-time data collection tools. During these visits, workers educate residents, eliminate breeding sites, and identify properties that pose a significant risk due to high mosquito production potential. Spatial risk mapping has identified “priority properties” and roadside storm catch basins requiring repeated interventions. As of late 2025, *Ae. aegypti* detections remain geographically confined to the Larnaca district, with no confirmed records from other parts of Cyprus.

Integrated control measures, including preparedness for the application of SIT aim to contain spread and reduce population densities of invasive *Aedes* species. These actions are implemented within national public health programs and under the guidance of the International Atomic Energy Agency (IAEA). Public engagement and citizen participation are considered increasingly important, especially as *Ae. albopictus* continues to spread island wide [137,138,139].

### 2.2. Traditional Control: Characteristics and Issues

Traditional mosquito control strategies (Figure 2) remain the cornerstone of integrated vector management programs across Europe. These approaches, which include source reduction, trapping, biological control agents, and chemical interventions targeting both immature and adult mosquitoes, have been refined over decades of operational experience. However, in many European countries, mosquito control is still mainly based on chemical adulticide treatments.

Mosquito control efficacy is increasingly challenged by insecticide resistance, environmental concerns, and the complex urban landscapes where invasive *Aedes* species thrive.

#### 2.2.1. Source Reduction

Source reduction, also known as environmental management or habitat modification, constitutes the most fundamental and sustainable approach to mosquito control. This strategy focuses on eliminating or modifying aquatic habitats that serve as mosquito breeding sites, thereby preventing larval development at its source. For container-breeding species such as *Ae. albopictus* and *Ae. aegypti*, which exploit small artificial water-holding containers in urban and peri-urban environments, source reduction is particularly critical.

The principle underlying source reduction is straightforward: without suitable oviposition and larval development sites, mosquito populations cannot establish or maintain themselves. In practice, this involves removing unnecessary water-holding containers, properly managing essential water storage, ensuring adequate drainage systems, and maintaining cleanliness of potential breeding sites. Common targets include discarded tires, flowerpot saucers, blocked roof gutters, cemetery vases, construction materials, and any artificial containers that can accumulate rainwater.

Community engagement (see Section 2.5) is essential for effective source reduction, as the majority of *Aedes* breeding sites are located in private properties. However, the labor-intensive nature of source reduction, the need for sustained community cooperation, and the continuous generation of new breeding sites in urban environments present ongoing challenges to this approach.

#### 2.2.2. Mass Trapping

Mass trapping strategies aim to reduce mosquito populations by deploying large numbers of traps to capture adult mosquitoes before they can reproduce or transmit pathogens. Unlike surveillance trapping, which focuses on monitoring mosquito presence and abundance, mass trapping seeks to achieve population suppression through sustained capture of sufficient numbers of individuals to impact the reproductive capacity of the target population.

Several trap designs have been developed and evaluated for mass trapping of *Aedes* mosquitoes. The BG-Sentinel trap, originally designed for surveillance, has been widely tested for mass trapping applications. This trap uses visual cues, convection currents, and chemical attractants (including CO_2_ when available) to lure host-seeking mosquitoes. Other trap types include oviposition traps that target gravid females seeking egg-laying sites, and the Gravid *Aedes* Trap (GAT), which combines an attractive dark surface with a sticky surface to capture mosquitoes.

A comprehensive three-year cluster randomized controlled trial conducted in France evaluated the efficacy of mass trapping for *Ae. albopictus* control in peri-urban communities [140]. The intervention combined passive oviposition traps and host-seeking traps with source reduction and larviciding. Results showed that mass trapping reduced mosquito abundance by 36–64% in some communities, though efficacy varied considerably based on local conditions, trap density, and house coverage. The highest reductions occurred with high trap density and extensive house coverage, emphasizing the importance of adequate spatial coverage for population-level impact.

GAT has shown particular promise for *Ae. aegypti* control. Semi-field assessments demonstrated that GAT recaptured 50–65% of released mosquitoes regardless of the number and size of breeding sites present, with capture rates comparable to BG-Sentinel traps [141]. Importantly, GAT successfully captured gravid females, though oviposition activity often occurred prior to capture, suggesting that source reduction interventions should precede trap deployment for optimal effectiveness.

Despite promising results in controlled settings, mass trapping faces several operational challenges. The approach requires substantial initial investment in trap procurement, ongoing costs for trap maintenance and monitoring, and sustained deployment over extended periods to achieve meaningful population suppression. Additionally, trap effectiveness can be influenced by mosquito behavior, competing hosts and oviposition sites, and environmental factors. The third year of the French trial revealed particular challenges in sustaining community participation, which significantly impacted overall effectiveness, highlighting the importance of continued engagement and support for community-based interventions [140].

#### 2.2.3. Biological Control

Biological control employs natural enemies of mosquitoes—including predators, parasites, and pathogens—to reduce vector populations in an environmentally sustainable manner. This approach offers several advantages over chemical control, including target specificity, absence of chemical residues, and reduced likelihood of resistance development. However, biological control agents must be carefully evaluated for efficacy, safety, and compatibility with other control methods before widespread implementation, furthermore it is important that native biological control agents are used.

*B. thuringiensis* subsp. *israelensis* and *Lysinibacillus sphaericus* (formerly *Bacillus sphaericus*, Bs) represent the most widely used and successful microbial control agents for mosquito larvae [142,143]. The high target specificity of Bti, combined with its excellent safety profile for non-target organisms and humans, has made it the biological larvicide of choice worldwide.

Formulations combining Bti and Bs have demonstrated enhanced efficacy and persistence compared to either agent alone. A field evaluation in northeastern Italy showed that a granular Bti + Bs formulation achieved high reduction in *Cx. pipiens* s.l. larval abundance in highly vegetated ditches—the primary rural larval habitats for this WNV vector [144]. The study observed a 93% reduction in larval abundance 24 h post-treatment, with effectiveness against larvae persisting up to 22 days and a residual effect of 99.5% maintained for 28 days. Notably, pupal density reduction exceeded 98% from days 14 to 28 post-treatment.

Recent large-scale operational data from northeastern Italy, encompassing over 30,000 catch basins inspected from 2019 to 2021, provided valuable insights into the real-world performance of larvicides [145]. The study revealed that approximately 5% of catch basins contained live late-stage larvae and/or pupae following treatment. Importantly, the intervals between treatments and inspections significantly influenced efficacy, with opposite associations observed for diflubenzuron (negative association) versus Bti + Bs (positive association) treatments, likely reflecting their different modes of action. For Bti + Bs treatments specifically, the percentage of positive catch basins for *Ae. albopictus* increased from 2% on day 7 to 13% on day 21 post-treatment, suggesting that shorter treatment intervals are necessary for sustained control.

A randomized controlled trial in Switzerland evaluated different application frequencies of VectoMax FG (Bti + Bs combination) in urban catch basins targeting *Ae. albopictus* and *Culex* species [146]. Results demonstrated that suppression of all taxa peaked within 20–30 days post-treatment. Notably, *Culex* spp. exhibited persistent suppression exceeding 90% for up to 6 weeks, while *Ae. albopictus* maintained comparably high suppression levels for up to 4 weeks. The study concluded that reapplication at 4-week intervals provided effective (>90%) suppression for both species, with increased application frequency enhancing overall effectiveness and reducing variability in mosquito abundance.

Despite the numerous advantages of Bti-based products, concerns have emerged regarding potential non-target effects on chironomid midges (Diptera: Chironomidae), which are important components of aquatic food webs. A comprehensive study in German wetlands examining the ecological consequences of Bti application at operational mosquito control rates found that chironomid larvae were the most affected non-target organisms in Bti-treated pond mesocosms [146]. The application of operational Bti field rates reduced overall chironomid emergence rates to approximately half of control rates, with effects observed across artificial mesocosms and realistic field conditions. These findings suggest that large-scale applications of Bti in seasonal wetlands, particularly within protected areas such as national parks and nature reserves, should be carefully considered to minimize unintended ecological impacts.

Predatory fishes, particularly larvivorous species such as *G. affinis*, *G. holbrooki*, and *Poecilia reticulata*, and copepod crustaceans can be effective predators of mosquito larvae. While the use of *Gambusia* species is no longer consented in Europe due to their invasiveness [147], research on native copepod species has shown encouraging results. The cyclopoid copepod *Megacyclops viridis*, field-collected in Germany, exhibited high predation efficiency against first-instar larvae of *Ae. albopictus* under both laboratory (up to 96%) and semi-field conditions (65.7%) [148]. However, the practical implementation of copepod-based biological control faces several challenges. The limited prey range (primarily first-instar larvae), variable predation rates across mosquito species, need for mass production systems, and questions regarding long-term establishment and persistence in diverse container habitats require further investigation before widespread operational deployment.

#### 2.2.4. Chemical Substances

Chemical larvicides target immature mosquito stages in their aquatic habitats, preventing adult emergence and thereby reducing the potential for disease transmission. Several classes of larvicides are currently available, each with distinct modes of action, efficacy profiles, and environmental characteristics.

Temephos, an organophosphate insecticide, has been widely used for mosquito larval control in drinking water containers and other habitats. However, extensive use has led to widespread resistance in many mosquito populations globally, limiting its continued effectiveness [149]. Similarly, carbamate insecticides such as bendiocarb have been employed for larval control, though resistance has also been documented in several vector species [150,151,152].

Insect growth regulators represent a distinct class of larvicides that interfere with normal insect development rather than directly killing larvae through neurotoxic or other acute mechanisms. The two most used IGRs in mosquito control are methoprene (juvenile hormone analog) and diflubenzuron (chitin synthesis inhibitor), with pyriproxyfen emerging as an increasingly important alternative.

Methoprene mimics natural insect juvenile hormone, preventing metamorphosis and causing mortality during the pupal stage or preventing adult emergence. While generally effective against many mosquito species, resistance to methoprene has been detected in some populations, particularly *Culex* species.

Diflubenzuron inhibits chitin synthesis, an essential process for cuticle formation during molting. It is effective against a broad range of mosquito species and has been extensively used in catch basin treatments. However, alarming levels of diflubenzuron resistance have emerged in *Cx. pipiens* s.l. populations in Europe. In northern Italy, resistance to diflubenzuron reached extraordinary levels, with some populations exhibiting up to 2900-fold resistance associated with mutations (I1043M and I1043L) in the chitin synthase gene [145]. Spatial analysis revealed repeated diflubenzuron treatment failures against *Cx. pipiens* s.l. in areas of the Venice lagoon, where the highest frequencies of resistance alleles have been reported. More recently, the I1043F mutation was detected in Crete, confirming the geographic expansion of diflubenzuron resistance across Europe. These findings seriously threaten the long-term viability of diflubenzuron, one of the few larvicides still approved under European biocide regulations.

Pyriproxyfen, a juvenile hormone analog with a pyridine-based structure, affects mosquito morphogenesis, reproduction, and embryogenesis [153]. The morphogenetic effect is primarily observed during larval-pupal transformation, resulting in death at the pupal stage with failure of adult emergence [154]. Pyriproxyfen exhibits high activity against mosquito larvae at very low dose rates (ppb to low ppm range), has low mammalian toxicity (oral LD_50_ > 5000 mg/kg in rats), and demonstrates minimal environmental impact when used appropriately [145]. Laboratory studies have shown that even organophosphate-resistant mosquito strains pressured with pyriproxyfen for multiple generations did not develop increased tolerance, suggesting a lower propensity for resistance development compared to conventional insecticides [155]. However, resistance to pyriproxyfen has been recently reported in a wild *Ae. albopictus* population [156]. Pyriproxyfen formulations are effective against multiple vector species, including *Ae. aegypti*, *Ae. albopictus*, *Cx. pipiens* s.l. and *Cx. quinquefasciatus* [157,158,159]. A unique property of pyriproxyfen is its potential for autodissemination [160]: contaminated adult females or males can transfer the compound to oviposition sites, causing effects on egg eclosion and inhibition of emergence in subsequent generations [161], though this mechanism requires further field validation (See Section 2.3.1).

Monomolecular surface films (MSF), such as those based on silicone compounds (e.g., Aquatain), create a physical barrier on the water surface that prevents mosquito larvae and pupae from accessing atmospheric oxygen, leading to hypoxia. A study in northeastern Italy evaluated Aquatain’s efficacy against *Ae. albopictus* and *Cx. pipiens* complex in catch basins [162]. While the product was effective in reducing emerging adults for both species, its duration was significantly affected by rainfall. Intensive showers (>10 mm daily) reduced efficacy, with increased adult emergence observed after approximately 2 weeks, suggesting that climatic factors must be considered when determining reapplication timing. Opposed to the biological control products aimed for larviciding, the advantage of the MSF application is that these products suppress mosquitoes while they are in the pupal stage [163].

Adult mosquito control through insecticide applications, commonly referred to as adulticiding, targets flying adult mosquitoes and represents a rapid intervention tool, particularly during disease outbreaks or when adult mosquito population nuisance reaches intolerable levels. Adulticides can be applied through various methods, including ground-based ultra-low volume (ULV) spraying, thermal fogging, indoor residual spraying (IRS), and targeted space treatments.

Synthetic pyrethroids constitute the most widely used class of adulticides for mosquito control globally, including in European operational programs. These compounds—including permethrin, deltamethrin, cypermethrin, and lambda-cyhalothrin—are neurotoxic insecticides that target voltage-gated sodium channels, causing rapid knockdown and death of mosquitoes. Pyrethroids offer advantages including high insecticidal activity at low doses, rapid knockdown effect, relatively low mammalian toxicity, and photodegradation that limits environmental persistence. However, the extensive and repeated use of pyrethroids has driven the evolution of resistance in multiple mosquito species of epidemiological importance. The operational implications of pyrethroid resistance are significant. During the largest West Nile virus outbreak in southern Spain in 2024, emergency vector control operations included focal adulticiding with cypermethrin alongside Bti-based larviciding [126]. While adult mosquito activity persisted into late autumn, the study highlighted the critical importance of rapid, coordinated expert interventions combining multiple control modalities to manage disease outbreaks.

Organophosphate insecticides such as malathion and fenitrothion act as acetylcholinesterase inhibitors, causing overstimulation of the nervous system. While historically important in mosquito control, organophosphates are being phased out in many regions due to higher mammalian toxicity compared to pyrethroids and increasing resistance.

### 2.3. Genetic Control: Characteristics and Issues

Genetic control (Figure 2) involves using different techniques to control pest or vector populations by the release of modified conspecific insects. This approach includes: (i) the repeated release of sterile males to abate the reproductive potential of the target species and gradually reduce the number of individuals [164,165]; (ii) the introduction of genes capable to spread and reduce the harm associated with the target species [165,166,167]. Common to all genetic control strategies is the lack of any effect on non-target species and the environment because both sterile males and modifying genes only exercise their action against conspecific individuals.

Suppression approaches exploit phenomena of egg sterility mediated by the released males that can be either natural or artificially induced. Males can be sterilized by a pre-release treatment that is usually operated with ionizing radiations (mainly gamma- or x-rays) to damage reproductive cells leading to inviable or highly defective sperms (sterile insect technique = SIT) [168,169]. As an alternative, a functional but not absolute sterility can be induced by the exploitation of symbionts capable to modulate cytoplasmic compatibility between sperms and oocytes at the moment of the fertilization so that only males and females harboring the same strains of the bacterium are able to reproduce successfully (incompatible insect technique = IIT) [170]. This is the case of the bacterium *Wolbachia* that is a quite common endosymbiont of insects, other arthropods and nematodes [170,171,172] and that shows this capability both in native and artificially infected species [170]. A third option to obtain males capable of inducing egg sterility is the recourse to genetic modification approaches [173,174]. Further suppression strategies are based on gene drive systems aiming at using released males to spread lethal genes with mating. These genes may induce the mortality of the larvae produced by crosses with wild-type females [175,176] or cause female progeny to be sterile [177].

Despite effectiveness and ecocompatibility, a series of issues still limit the implementation of genetic control strategies addressed to population suppression. Indeed, suppression approaches are self-limiting control strategies as effects rapidly drop after the interruption of the releases (unless eradication is achieved). Furthermore, these approaches can be deployed only against a number of vector species because not all vectors are equally suitable for laboratory colonization, mass production and efficient sexing. In the case of *Wolbachia*, additional limiting factors are that not all species are suitable for the infection of this bacterium and that sexing must be sufficiently accurate to avoid any undesired spread of female individuals harboring the artificial *Wolbachia* infection [170,172]. At last, large scale and long-term control programs require significant investments, including those necessary for building a dedicated biofactory. This issue makes a cost–benefit analysis fundamental before the implementation of any suppression program against a vector species [170].

Population modification approaches aim at reducing the vectorial capacity of a target population by favoring the spread of useful genes affecting vector competence or vector survival. These genes can be naturally present in certain populations or can be artificially introduced in a species, and they can be part of the nuclear DNA or can be carried by transposons or heritable endosymbionts [165,178]. These strategies include the exploitation of *Wolbachia* strains that are capable of spreading throughout a natural population (thanks to the CI) and blocking pathogens [165,170,178,179], or gene-drive mechanisms coupled with transmission-blocking gene constructs [177,180,181].

Differently from population suppression strategies, useful genes that are naturally capable of spreading can guarantee population modification approaches to be self-sustaining [182]. This means that, once successfully ignited, these strategies do not require further releases of insects, but managing any undesired side effect could be more challenging [170]. Nevertheless, the population replacement strategy (PRS), that involves exploiting *Wolbachia* to replace wild competent vector populations with conspecific populations incapable of transmitting viruses, is undoubtedly the most successful and widely used genetic control strategy addressed to mosquito vectors [170]. In fact, this approach has already achieved the result of eliminating or strongly reducing the autochthonous cases of dengue in certain experimental areas [183]. Other population modification approaches that are based on genetic modification are under testing in the countries where this kind of application is already regulated and consented [184].

#### 2.3.1. SIT in Europe

SIT has emerged as a sustainable, environmentally friendly tool for suppressing insect populations and is increasingly incorporated into integrated pest and vector management programmes worldwide [185]. Originally designed for area-wide eradication of agricultural pests, SIT is now applied more broadly for population suppression, including in mosquito control, attracting growing interest from public health authorities and commercial producers. As SIT adoption expands, the need for consistent regulatory frameworks, quality assurance, and risk assessment has become evident. Current evidence indicates that SIT based on irradiation can be implemented safely within existing regulatory systems [185].

Recent experience has also shown that successful mosquito SIT programmes depend not only on technical performance but on strong community engagement, transparent communication, careful site selection, and integration into broader vector management frameworks [169]. Designing effective field trials requires stakeholder mapping, local adaptation of procedures, high-quality mass-release systems, and robust monitoring methods to evaluate impacts and operational benefits [168]. These principles have guided the progressive development of SIT activities in Europe over the past two decades.

The first European SIT pilot trials were conducted in Rimini (Emilia-Romagna Region, Italy) in 2004 and involved mass-reared, gamma-irradiated *Ae. albopictus* males resulting in a reduction in both *Ae. albopictus* egg density and fertility [184]. These encouraging results led to further experimentations contributing important insights into feasibility, community acceptance, and operational performance. Similar trials in the same Italian region demonstrated that sterile males released at the rate of 896–1590 males/ha/week induced a significant sterility level in the local population and that a 70–80% reduction in egg fertility usually corresponded to a significant decrease in the egg density in the study area [186]. SIT suppression trials were subsequently conducted in urban areas of the Valencian region (Spain) and were shown to reduce the adult and the egg population by 70–80% compared to the control area [187].

Greece experienced successful SIT trials against *Ae. albopictus* in Vravrona (Attica Region) where, in 2018 and 2019, weekly applications of 2200–3000 sterile males per ha resulted in elevated egg sterility within 5- and 10-ha sites, demonstrating promising suppression potential and justifying larger-scale trials [188,189]. The open field deployment was anticipated by a prerelease door-to-door campaign that helped raise public awareness, reduced breeding sites, and strengthened community involvement—key conditions for SIT success [190].

Large scale SIT trials (over 45 ha and the entire mosquito season) integrated with routine IVM, were also recently conducted in Morcote (Switzerland), highlighting a significant effect on egg counts, egg hatching, and female density compared to control [191].

More recently, boosted-SIT trials, combining sterilized males with pyriproxyfen coating, were tested both in Spain and Greece demonstrating the possibility of integration of SIT with other control measures to enhance suppression effectiveness [192,193].

Being sustainability one of the major issues limiting SIT implementation, open field trials were usually accompanied by laboratory studies to improve SIT framework efficiency in terms of increased sterile male yield, increased male fitness, decreased female contamination among released mosquitoes, and more efficient and safe transportation [194,195,196].

#### 2.3.2. *Wolbachia* and the IIT Approach in Europe

Several IIT trials have been conducted worldwide, and a number of operational programs are currently active especially in tropical climate areas [170] but this strategy has had only limited application in Europe until now. The first European IIT open field trials were conducted in 2018 and 2019 in Rome to test, at a small scale, the capacity of this approach to reduce the mean egg fertility of an urban population of *Ae. albopictus*. For this purpose, the males of an *Ae. albopictus* line harboring *Wolbachia w*Pip instead of the native infection and fully incompatible with wild females were used [197,198]. Results were encouraging since at incompatible:wild ratios of, respectively, 0.7:1.0 and 1.1:1.0, a 16% and 35% reduction in the *Ae. albopictus* egg fertility was achieved, despite the study area being open to incompatible male dispersal and immigration of already mated females [199,200]. However, due to the small scale of the experiments, results are still not sufficient to recommend the use of this control strategy in Europe because of the lack of sufficient evidence of entomological effectiveness, i.e., reduction in the number of adult mosquitoes and, consequently, reduction in the biting rate in the target area [201].

### 2.4. Smart Technologies Supporting Surveillance and Control

Various smart technologies are starting to be integrated into strategies to prevent and fight mosquito-borne diseases by supporting surveillance and more targeted and sustainable control interventions (Figure 2).

Traps for monitoring can be equipped with smart sensors to identify and differentiate between mosquito species in real-time [202,203]. These traps may combine IoT devices, high-resolution cameras, and advanced Machine Learning (ML) algorithms for insect detection and classification to help in recognizing invasive species and furnish useful information regarding their behavior and phenology [204].

The use of drones equipped with multispectral cameras could support surveillance activities by contributing to the identification of breeding sites or mapping micro-environmental composition even in areas not easily reachable by humans [205].

Advanced technologies for monitoring enable the transformation of provided data into informative graphs and risk maps with multiple analytical perspectives. This approach maximizes the potential of the strategies and identifies vulnerable areas that might otherwise escape researchers’ notice [206]. The integration of complex, multi-domain data coupled with automation that these technologies can provide facilitates the early detection of various health threats, such as vector-borne disease outbreaks and its epidemiology. ML algorithms provide a robust framework for storing and processing the vast array of collected data, enabling more accurate and timely predictions, and a deeper understanding of the situation that opens new targets that were unseen before [207]. In this context, AI is already used as a means to develop models to predict mosquito distribution and estimate epidemic risk [208,209].

Regarding vector control, novel classic artificial intelligence (AI)-based methods for evaluating the potential insecticidal activity of unknown functional compounds are starting to be implemented in smart agriculture [210] and similar AI-based methods could also be applied to develop new control tools.

Automation and AI are already demonstrating how to enhance safety and efficiency of SIT and IIT frameworks by, as already mentioned [211], contributing to the development of highly efficient protocols for sexing but also supporting the setup of effective and sustainable release protocols [212].

Drones could also support control strategies as an example by applying larval source management activities or deploying sterile males or any genetically modified agents [213,214].

### 2.5. The Role of Communities in Mosquito Control

Citizen science refers to the active involvement of non-expert individuals in one or more phases of the scientific method, including data collection, analysis, and hypothesis testing (Figure 2) [215]. Unlike political leadership, community networks are stable over time, making them key allies in sustaining and sustaining public health and scientific initiatives.

Citizens are directly exposed to the burden of mosquito-borne diseases and can contribute to both source reduction (see Section 2.2.1) and surveillance (see Section 2.1). Community engagement, when integrated with vertically structured centralized approaches, can enhance the effectiveness, short-term success, and long-term sustainability of vector control programs [216,217]. This collaboration may range from individual participation to coordination between national authorities and small municipalities and should involve communities not only during implementation but also in project design and development from the outset [217].

To strengthen public health systems, citizen collaboration should be combined with emerging technologies in data analytics and predictive artificial intelligence (AI) models [218]. Among early and successful examples, Mosquito Alert stands out as a pioneering initiative integrating citizen participation and digital surveillance. Launched in 2104 as a mobile application, it enables users to upload photographs of suspected Asian tiger mosquitoes. Analyses of data from 2014–2015 indicated that citizen participation reduced the surveillance costs by up to eightfold compared with traditional methods [219]. Beyond data collection, the project trained participants to identify different mosquito species using simple traits and raised awareness of vector-borne risks, fostering trust between citizens, scientists, and public authorities. By correcting for sampling biases inherent to mobile-collected data, Mosquito Alert demonstrated that citizen-generated information has qualitative and predictive power comparable to conventional surveillance systems [220], supporting the feasibility of long-term, cost-effective monitoring strategies [219].

Building on similar principles, other initiatives have emerged, including Carney [221], which integrated Mosquito Alert data with iNaturalist and GLOBE Observer’s Mosquito Habitat Mapper and Land Cover applications. In parallel, citizen contributions to biodiversity surveillance have expanded rapidly; in 2020, approximately 74.5% of insect observations in the Global Biodiversity Information Facility (GBIF) were provided by citizens [222] and the number of collaborative platforms continues to grow [223].

Community involvement is also central to intervention-based strategies, as exemplified by the World Mosquito Program, which aims to control arboviral diseases by replacing *Ae. aegypti* populations with *Wolbachia*-infected mosquitoes incapable of transmitting viruses [224]. The program relies on strong public support and collaboration with local associations to ensure successful implementation. When initiatives integrate into local context, informed citizen participation improves information flow and data quality. While data collection remains the most common form of collaboration [225], community cultural knowledge and lived experience can also contribute to data interpretation and decision-making. Given disparities in internet access and technological development, citizen science approaches must be adapted respectfully to local contexts. When effectively engaged, communities can expand monitoring to underrepresented areas, refine analysis, and help prioritize intervention zones [226].

Citizen participation can also mitigate research bottlenecks related to limited resources or time-intensive methodologies, while fostering a sense of ownership and trust in the scientific process. This empowerment supports broader dissemination of project objectives, sustained engagement, and resistance to misinformation [227].

Conducting KAP Knowledge, Attitude, and Practice surveys (KAP) represents a key tool for assessing community awareness and behaviors related to mosquitoes and MBDs in Europe, where transmission is generally sporadic [228,229]. These surveys assess knowledge of mosquito breeding sites, perceived disease risk, and preventive behaviors, informing the design of effective interventions. European KAP studies reveal substantial variability in MBD awareness, influenced by geographic location, outbreak history, education, socioeconomic status, and gender [190,229,230,231,232]. These findings highlight the need for targeted education and community engagement strategies. KAP questionnaires therefore provide critical insights into behavioral and knowledge gaps shaping preventive practices and informing efforts to reduce mosquito proliferation and MBD emergence across Europe [233].

### 2.6. Cost-Effectiveness of Mosquito Control

Concurrently with the increasing health burden, the economic and social impacts are rapidly increasing across Europe and the Mediterranean Europe. Despite this growing impact, the economic costs of mosquito-borne disease in Europe remain substantially underreported and underestimated, regarding medical damage, losses and the comprehensive costs of prevention and vector control. We recently reported that the cumulative global economic cost of *Ae. aegypti* and *Ae. albopictus* and their associated diseases exceeded US $94.7 billion over 45 years (1975–2020), rising to more than US $300 billion when long-term consequences, such as chronic osteoarthritis following chikungunya infection or neurological impacts of Zika, are included [234]. European data represent only a fraction of these estimates, being greatly underreported, masking the growing financial burden of Mediterranean countries and therefore limiting the ability to anticipate budgetary needs and prioritize proactive investment on prevention.

Globally, reported economic costs of medical and non-medical damages and losses due to mosquitoes and their diseases are ten times higher than investment in management, equating to a more than ten-fold difference in terms of annual and total costs of damages vs. management [234]. Clearly, the investment on management increased at a much lower rate than the increasing economic burden, reflecting an uneven and limited investment on mosquito control, remaining largely reactive in response to outbreaks, fragmented and uneven across Mediterranean regions. Recent interdisciplinary evidence from France shows that even limited chikungunya and dengue outbreaks can trigger wide-ranging health, economic, and social consequences that extend far beyond reported case numbers and economic costs [235]. The last years of massive chikungunya and dengue autochthonous transmission confirmed that emergency interventions places regional health agencies and mosquito control operators at risk of saturation when cases accumulate, especially during summer periods when staffing is reduced and healthcare demand is already high. So, the chronic underfunding of epidemiological surveillance and vector control represents a critical vulnerability as, when surveillance and control systems are overwhelmed, transmission may escalate rapidly, generating cascading and ripple effects across healthcare, economic activity, education and society, exacerbated by illness-related absenteeism and media coverage [235,236]. Reliance on emergency chemical interventions risks environmental concerns, public opposition and declining effectiveness due to insecticide resistance, while also increasing operation malfunctioning during wide-area outbreaks. Important benefits such as reducing healthcare and mosquito control saturation, protection of tourism revenues, mitigation of social disruption, reduced absenteeism and related productivity effects and reduced inequality amplification are not captured in conventional evaluations [235]. Importantly, early action with proactive (preventive) strategies rather than reactive (emergency) responses or even inaction will be more cost-effective [237,238,239,240].

Alone, greater financial investment is insufficient. Control programs frequently suffer from lack of quantitative evidence of effectiveness, ineffective implementation, inadequate coverage, operational weakness and increasing insecticide resistance [240,241]. Rather than generalized estimates, multiple studies in different areas are needed, as the cost-effectiveness of mosquito control is highly context-dependent and varies among ecological, epidemiological and cultural settings. Costs depend on the scale of implementation, typically decreasing as the targeted area and population increase due to economies of scale. Evidence from *Wolbachia* deployments suggests that costs per km^2^ tend to increase in densely populated urban areas, reflecting greater operational complexity [242]. However, the cost per person covered decreases as population density increases, since more individuals benefit from the intervention, resulting in economies of scale. In addition, cost-effectiveness studies of management strategies with statistically significant positive findings favorable to the intervention under study are also more likely to be published, creating a potential publication bias [243].

In Europe, more research is urgently needed, as current evidence on the effectiveness and economic efficiency of vector control strategies remains limited and fragmented [238,240,244,245]. In the recent report of the European Mosquito Control Association (EMCA) about the effectiveness of mosquito control in Europe [201], there is a marked absence of high-quality published data on the effectiveness of interventions, and the data about cost-effectiveness is particularly scarce [246,247,248,249]. Therefore, there is a critical need for robust, case-by-case cost-effectiveness evaluations of strategies reducing mosquito densities and preventing arboviral disease transmission.

Worldwide, cost-effectiveness studies of conventional vector control strategies are scarce, related to larval control [250], source reduction [251], community participation [252] and few innovative techniques as SIT [253]. A growing body of literature has evaluated the cost-effectiveness of *Wolbachia*-based population replacement strategies for dengue in several countries, finding evidence of the cost-effectiveness of this strategy [242,254,255]. A clear benefit is that it generates long-term reductions in transmission without continuous releases, in contrast with population suppression strategies such as SIT or IIT, that require sustained funding.

Currently, the evaluation of effectiveness, the economic costs and the cost-effectiveness lack consistency and standardization. It is important to clarify how the cost-effectiveness is density-dependent, varied among the different settings, and in particular related to the baseline vector abundance and the quality of the implementation [241]. Analyses should clearly specify the adopted perspective—healthcare provider, health sector, or societal—with particular attention to societal costs, including productivity losses and non-medical expenses, which are especially relevant in European welfare systems [256,257]. Importantly, broader economic effects, such as impacts on tourism and regional development in Mediterranean areas, remain largely unquantified and warrant inclusion in future assessments [258,259]. From a European and Mediterranean policy standpoint, several priorities emerge: (i) harmonizing economic evaluation methods across countries; (ii) improving surveillance-linked costing of vector control interventions; (iii) investing in proactive, preventive strategies rather than emergency responses; and (iv) supporting innovation and scale-up of cost-effective, environmentally sustainable tools. Mathematical modelling should play a central role in optimizing intervention design, timing, and spatial targeting under diverse European contexts. Low awareness among the public and healthcare professionals, fragmented responsibilities, inadequate media information and insufficient long-term funding are identified as the main preparedness gaps. Ultimately, strengthening the economic and effectiveness evidence base for mosquito control is essential to support efficient, sustainable, and integrated strategies aligned with EU health security, climate adaptation, and One Health objectives. Beyond economic gains, such strategies would deliver substantial public health benefits, reducing disease burden, protecting vulnerable populations, and enhancing societal well-being across the Mediterranean region.

## 3. Perspectives and Conclusions

Climate change, urbanization, and global mobility are reshaping the landscape of vector-borne disease (VBD) risk across Europe, accelerating the spread, establishment, and seasonal persistence of invasive mosquito species. Modelling studies for Greece and Italy indicate that projected temperature and precipitation shifts will create increasingly favorable conditions for IMS establishment by mid-century, with spatial risk maps showing a clear expansion of climatically suitable areas [260]. These projections align with recent real-world incursions of *Ae. aegypti* and *Ae. albopictus* in Cyprus, which triggered coordinated emergency responses involving intensified surveillance, field verification, and the development of rapid action and contingency plans to prevent further spread [137]. At the same time, exceptionally warm winters, such as the record-breaking December 2022 temperatures observed in Greece, may weaken seasonal barriers that traditionally limit vector activity, enabling unusual patterns of overwintering and prolonging transmission seasons [261].

Restriction of certain biocides in Europe under the Biocidal Products Regulation (BPR, Regulation (EU) 528/2012) has also created significant challenges, primarily driven by the ambitious goal of increasing human and environmental safety, which has led to high regulatory hurdles, reduced product availability, and increased market unpredictability.

Furthermore, general practitioners in the public health system are often not sufficiently prepared to deal with little-known diseases that are not endemic to Europe and are incapable of promptly recognizing mosquito-borne diseases that require specific treatment.

**Figure 3:**
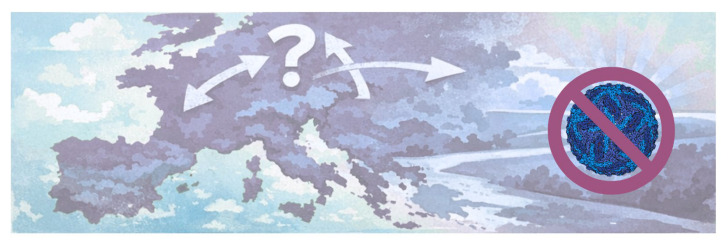
Addressing the emerging risks associated with mosquito-borne diseases.

Collectively, these developments highlight that Europe faces increasing VBD pressures while often remaining underprepared, with public health resources strained by competing priorities (Figure 3) [262,263]. Addressing these emerging risks requires proactive and co-produced One Health strategies that integrate climate-informed early warning systems, cross-sectoral adaptation planning, and decision-support tools capable of tracking climate-sensitive disease risks across hazard, exposure, and vulnerability domains [262]. Leveraging lessons from endemic regions, fostering regional partnerships, and prioritizing anticipatory rather than reactive approaches will be essential for strengthening Europe’s resilience against climate-sensitive VBD threats [263].

Given this context, the future of mosquito control against virus vectors must be increasingly driven by innovative and integrated vector management (IVM) approaches coordinated at area-wide level.

To face insecticide resistance issues, coordinated national and regional surveillance strategies are increasingly being adopted. As an example, in France, the Integrated Plan of Insecticide Resistance Surveillance (PSIR) combines sentinel site monitoring, standardized bioassays, molecular diagnostics, and a quantitative resistance risk stratification system to inform targeted responses [264]. This approach facilitates data-driven vector control and prioritization of alternative interventions.

The fixation of mutations conferring resistance can be limited by the insecticide class rotation, the incorporation of synergists such as piperonyl butoxide (PBO), and increased reliance on biological control strategies. In addition, strategic investment in next-generation insecticides with novel modes of action, also based on AI-tools, coupled with improved stakeholder education on resistance management, will be crucial to safeguarding vector control efficacy in Europe.

Despite the demonstrated effectiveness, the application of genetic control strategies is still limited by cost-effectiveness issues because translating experimental trials into operational programs requires large investments and long-term programming. This problem mainly regards suppression-based strategies that are strictly dependent on the continuous production and release of sterile males at area-wide level [170]. Estimating the costs due to the health-related issues determined by the vectors and comparing them with the investments that are necessary for prevention could encourage governments to support genetic control programs. At the same time, any innovative technology supporting genetic control sustainability can increase the chance of success of this approach. As an example, innovative AI-based technologies to achieve perfect sexing have recently become available [211] and can consent to increase the yield of males to be released out of the reared larvae and prevent any undesirable escape of vector females. Also, the application of the IIT could offer some advantages over SIT because incompatible males always induce full wild female sterility without requiring any treatment before release and compared to irradiated males showed fully preserved male mating competitiveness [265]. However, while SIT for insect control is already regulated in Europe, the experimentation of other genetic control strategies under open field conditions still requires a specific regulatory framework. With regard to *Wolbachia*-based control strategies, the Commission Implementing Decision (EU) 2018/1623 of 29 October 2018 [266] specified that *Wolbachia* bacteria or preparations containing such bacteria used to inoculate mosquitoes with the aim of creating artificially infected mosquitoes for vector control purposes shall be considered biocidal products within the meaning of Article 3(1)(a) of Regulation (EU) No. 528/2012. Instead, artificially infected mosquitoes, irrespective of the infection technique used, should not be considered biocidal products or treated articles [172]. This decision is paving the way to an easier authorization for field trials involving mosquitoes with modified *Wolbachia* infection, especially when both mosquito species and *Wolbachia* strains used are already common in the study area [199,200,267]. In this context, large-scale IIT trials will be fundamental to evaluating both effectiveness and sustainability of this vector control strategy, while specific authorizations will be needed before *Wolbachia*-based products could be made available on the market.

The integration of both SIT and IIT with other control measures, in particular the use of larvicides and the exploitation of the released males to disseminate insect growth regulators (IGRs) to the breeding sites could increase effectiveness and sustainability of the approach.

Gene-editing techniques are providing new resources to achieve male sterility or the reduction in the vector competence that could increase the weapons available for vector control. The European council is working on a legal framework to regulate genome-editing applications in crops by proposing to categorize plants obtained through the exploitation of new genomic techniques (NGTs), resulting from targeted mutagenesis and cisgenesis, into two distinct categories: ‘conventional-like’ and ‘genetically modified organism (GMO)-like [268,269]. A similar classification could also apply to insects in the future and consent gene-edited mosquitoes to be tested as possible additional options to be used for arboviral control.

Advanced tools, including gene drives, may enable more targeted and long-term interventions, while improved monitoring and modeling, using genetic markers, smart sensors and artificial intelligence, will allow real-time assessment of efficacy and better outbreak prediction.

Careful evaluation of ecological impacts and active community engagement will be essential to ensure both effectiveness and public acceptance. By integrating advanced technologies, ecological understanding, and social participation, future mosquito control strategies can offer sustainable and proactive solutions to mitigate current and emerging arbovirus risks.

Due to the complexity of the factors that contribute to the emergence and spread of arboviral diseases, exploiting a One Health approach can be vital to properly facing this threat [270,271]. Collaborative efforts between public health professionals, animal health professionals and environmental experts are key to trying to predict outbreaks of vector-borne diseases and act before they pose a threat to the population. But the One Health approach, beyond being a conceptual term, must be measurable in order to determine whether it truly drives meaningful change. Only then can we assess whether this vision genuinely leads to improved quality and effectiveness of health protocols. A comprehensive review of over 1800 articles revealed that the concept was often assumed to be fulfilled without supporting evidence or was applied based on subjective interpretation [272]. It is therefore essential to establish standardized criteria or metrics to ensure that One Health is not merely a theoretical ideal, but a validated and demonstrably effective approach [273,274].

## Data Availability

No new data were created or analyzed in this study. Data sharing is not applicable to this article.
